# Pediatric Primary Tuberculous Osteomyelitis of the Mandible Mimicking Parotitis

**DOI:** 10.7759/cureus.2071

**Published:** 2018-01-15

**Authors:** Raja Kalaiarasi, Chellappa Vijayakumar, Ramalingam Archana, Ramalingam Natarajan

**Affiliations:** 1 Otorhinolaryngology, Sri Lakshmi Narayana Institute of Medical Science, Puducherry, India; 2 Surgery, Jawaharlal Institute of Postgraduate Medical Education and Research (JIPMER), Puducherry, India.; 3 Preventive Medicine, Jawaharlal Institute of Postgraduate Medical Education and Research (JIPMER), Puducherry, India.; 4 Otolaryngology, Jawaharlal Institute of Postgraduate Medical Education and Research (JIPMER), Puducherry, India.

**Keywords:** tuberculosis, osteomyelitis, mandible, parotitis, lymphadenitis

## Abstract

Tuberculosis (TB) is a worldwide public health problem; however, primary tuberculous osteomyelitis involving the mandible is extremely rare. Here, we report a 14-year-old boy who presented with a recurrent, generalized swelling of the cheek in the right side, mimicking parotitis. Fine needle aspiration cytology (FNAC) from the swelling was inconclusive. Contrast-enhanced computed tomography (CECT) of the head and neck revealed an osteolytic lesion of the mandible with a surrounding abscess. An intraoral incisional biopsy of the tissue showed a granulomatous lesion. The patient was started on anti-tubercular therapy (ATT) for six months. Our patient’s presentation underscores the clinical difficulty in establishing a diagnosis and considering tuberculous osteomyelitis in the differential diagnosis.

## Introduction

Tuberculosis (TB) is a worldwide public health problem, which has a diverse mode of presentation; so, the diagnosis is difficult and delayed. In India, the incidence of TB in 2015 was 217 per lakh population and the mortality due to TB was 36 per lakh population. Pure primary tuberculous osteomyelitis involving the mandible is very rare. We report a case of tuberculous osteomyelitis of the mandible presented as a recurrent, generalized swelling of the cheek without evidence of any active pulmonary foci. On follow-up, the patient showed a complete resolution of the mandibular lesion after six months of anti-tubercular therapy (ATT). Our patient’s presentation highlights the clinical difficulties in establishing the etiology. The management is outlined and the reasons for the delay in diagnosis are highlighted.

## Case presentation

A 14-year-old boy presented to the otorhinolaryngology outpatient department with a history of gradually progressive swelling in the right side of the cheek for three months and pain on opening the mouth for one week associated with on-and-off mild fever. The patient denied any constitutional symptoms and had no cough during the past six months. The patient had similar episodes twice in the past one year, lasting for 10 days, which subsided spontaneously. There was no significant family history or contact history of TB. The patient was averagely built, moderately nourished, and afebrile at the time of examination.

**Figure 1:**
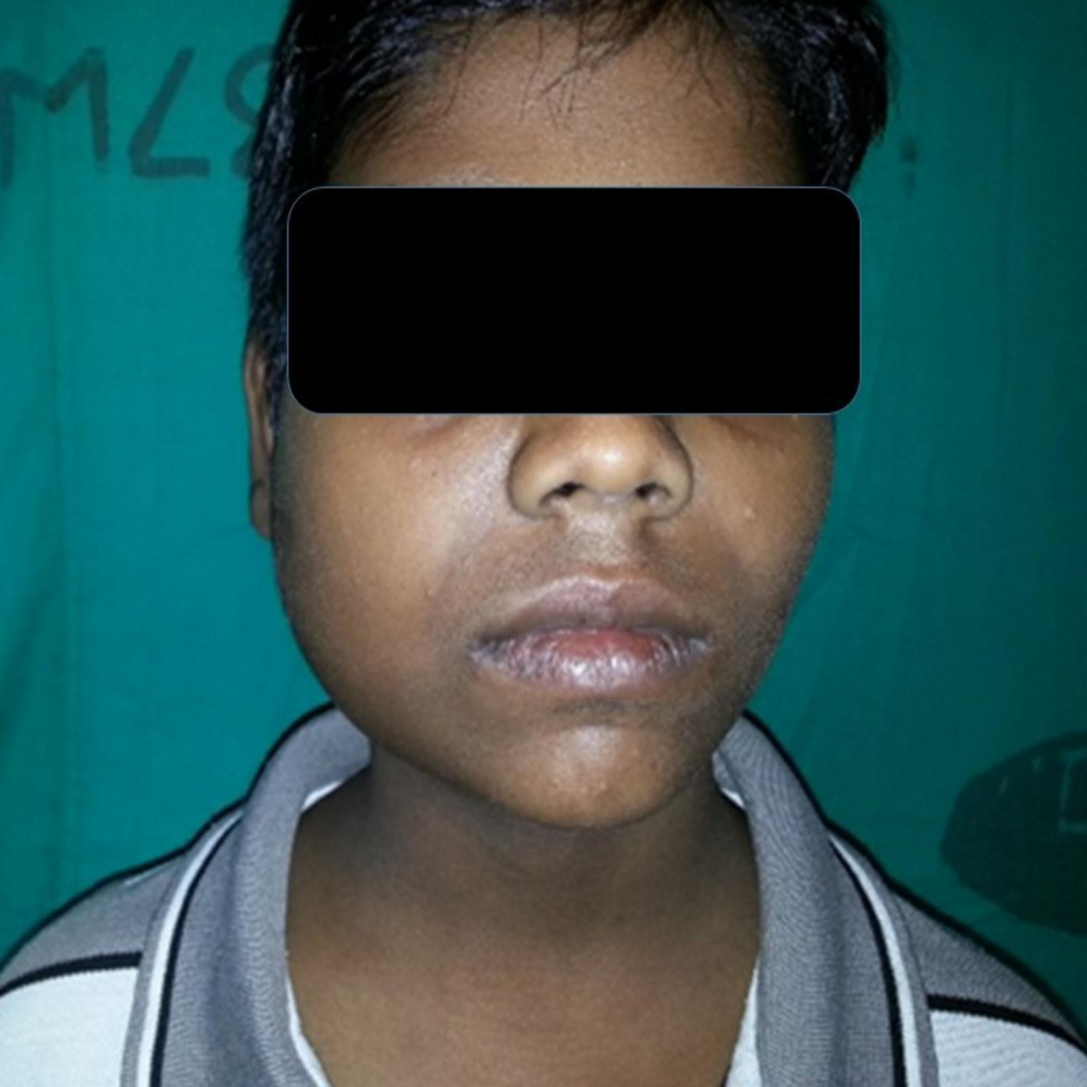
Diffuse swelling of the right side of the cheek with submandibular region involvement

Local examination revealed a firm, tender, non-fluctuant, non-pulsatile, diffuse, right-sided swelling of the submandibular and parotid region with normal overlying skin (Figure 1).

On an intraoral examination, the teeth were in good condition and there was no intraoral swelling or ulcer. Multiple, firm, 1.0 X 1.5 cm, enlarged, non-tender lymph nodes (the largest node measuring 2 X 2 cm) were palpable in the bilateral submandibular region. Respiratory system, cardiovascular system, and abdominal system examinations were normal. An ultrasound neck showed a diffusely enlarged right parotid gland with loss of normal echotexture and an enlarged submandibular gland. A provisional diagnosis of parotitis was made based on clinical history, regional swelling, and ultrasound features. The patient was started on intravenous antibiotics for seven days, but the patient did not improve well.

All hematological (Hb 12 gm/dl, total leukocyte counts - 6100 cells/cubic mm, differential leukocyte count: neutrophils - 74%, lymphocytes - 22%, eosinophils - 3%, and monocytes - 1%) and biochemical investigations (blood sugar - 92 mg/dL, blood urea - 10 mg/dL ) were within normal reference ranges except for increased erythrocyte sedimentation rate (ESR) of 60 mm in the first hour. The enzyme-linked immunosorbent assay (ELISA) test for the human immunodeficiency virus (HIV) and the surface antigen of the hepatitis B virus (HBsAg) were negative. The chest x-ray was normal.

**Figure 2:**
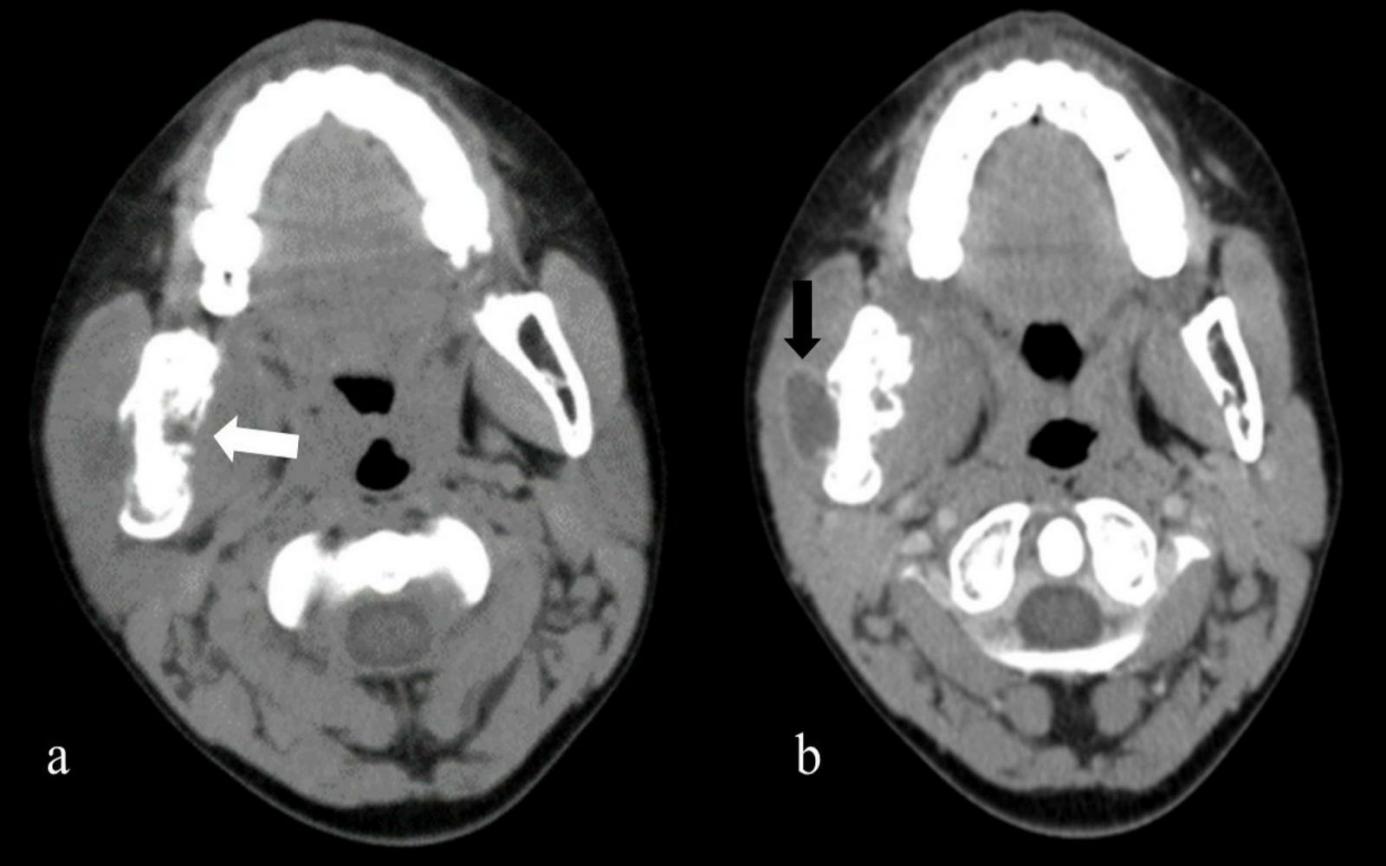
a) Contrast-enhanced computed tomography head axial view showing irregularly thickened cortex with loss of corticomedullary junction with multiple loosened areas showing a breach of the cortex and periosteal reaction (white arrow) with adjacent soft tissue component. b) Showing central hypoattenuation areas representing central necrosis (black arrow) with periosteal reaction

Fine needle aspiration cytology (FNAC) was performed on the swelling in view of the poor response to systemic antibiotics on day 10 of admission. However, FNAC was non-diagnostic. Since his symptoms did not improve, a contrast-enhanced computed tomography (CECT) of the head and neck with a 2-mm slice width was performed, which revealed an osteolytic lesion of the mandible with extension into the adjacent part of the body of the mandible. There was associated soft tissue swelling showing central hypodense areas with rim enhancement (Figure 2).

**Figure 3:**
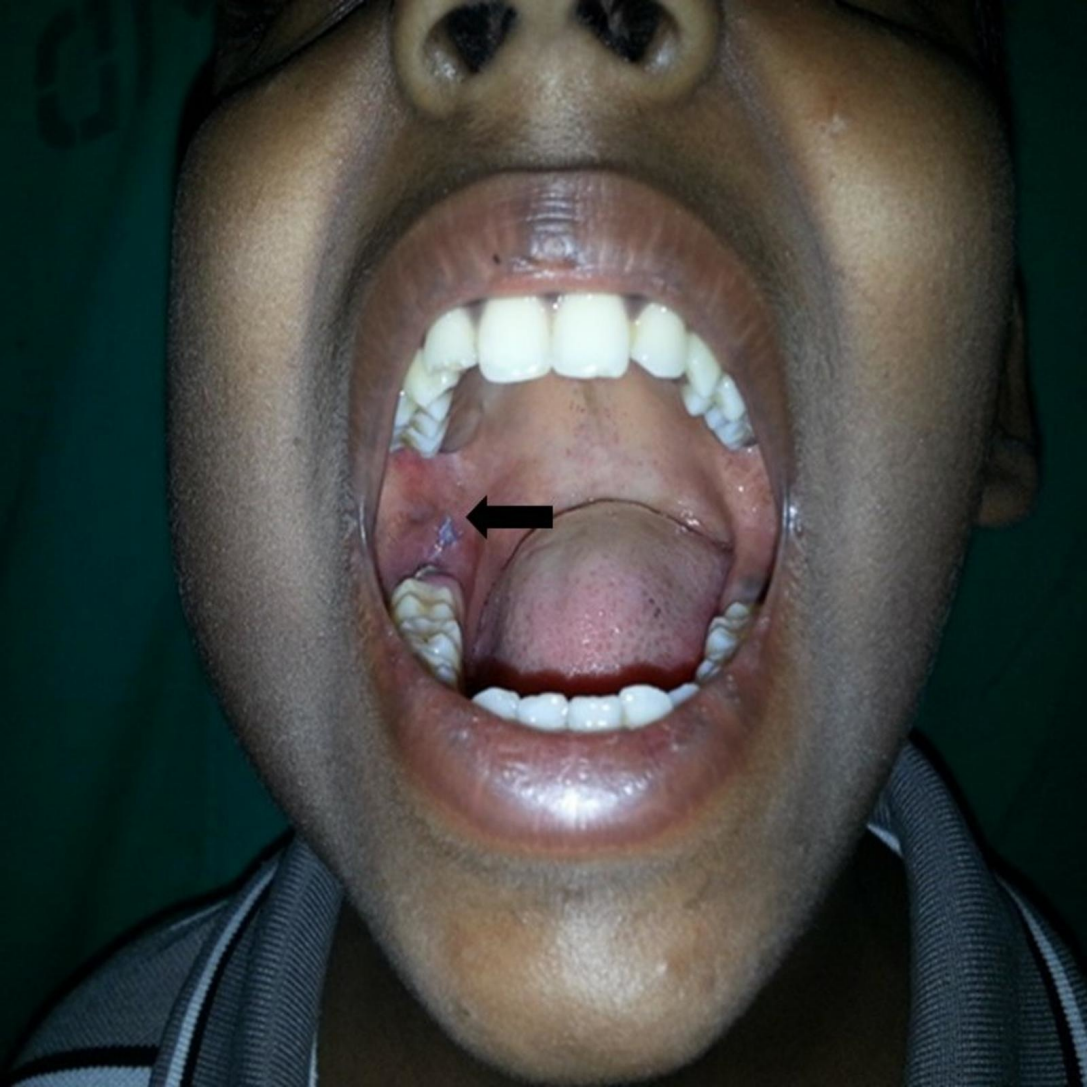
Intraoral photograph of the right mandibular region showing the biopsy site (black arrow)

The CECT findings were suggestive of lytic lesions of the mandible with a surrounding soft tissue abscess along with submandibular lymphadenopathy. The image morphology of the lesion was consistent with osteomyelitis. The Mantoux tuberculin skin test using five units of purified protein derivative was done keeping the suspicion of TB, but it was negative (induration size – 2x2 mm). So, an intraoral incisional biopsy of the tissue was taken behind the right second molar tooth (Figure 3).

**Figure 4:**
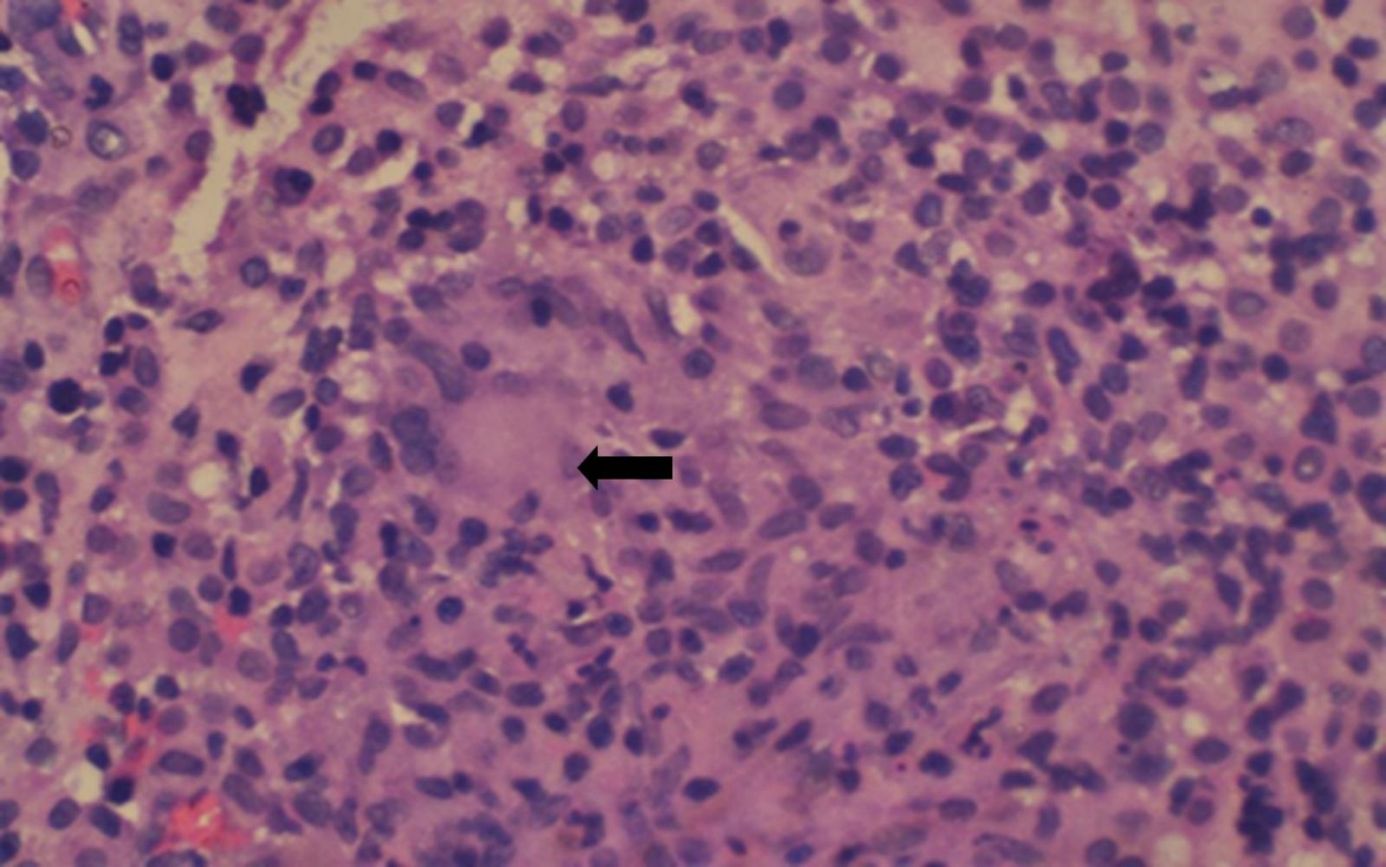
Histopathological picture (hematoxylin and eosin stain, 400x magnification) is showing an epithelioid cell granuloma with characteristic Langhans Giant cells (black arrow) surrounded by lymphocytes and plasma cells

A histopathology examination of the intraoral biopsy specimen revealed fibro-collagenous tissue with multiple epithelioid cell granulomas admixed with numerous plasma cells and occasional Langhans Giant cells (Figure 4).

Acid-fast staining for acid-fast bacilli was negative. These findings were strongly suggestive of TB mandible. The sputum examination was negative. Based on the above radiological and histological findings, a diagnosis of primary tuberculous osteomyelitis of the mandible was made. The patient was started on ATT for six months, and at the end of the six months, there was complete resolution of the swelling. Orthopantomogram (OPG) imaging was done after the completion of ATT, which was normal. If the OPG showed an abnormality, it warranted a repeat CECT of the head and neck to find out if any sequestra had formed within the mandible.

## Discussion

India accounts for one-fourth of the global incidence of TB, with nearly 2.8-million incident cases in the year 2015. Nearly 15%-20% of all TB cases occur in extrapulmonary sites. Among the extra-pulmonary sites, the most common are TB lymph nodes (35%) followed by pleural TB (20%) and TB in bones and joints (10%) [1]. Tuberculous osteomyelitis is very rare, constituting less than 2% of skeletal TB [1]. Mandible involvement is even rarer and usually affects older people [2]. In children, mandibular TB is highly unlikely, and very few cases have been reported in the literature [3]. The varied spectrum of presentation and the rarity of the condition often lead to a diagnostic dilemma and delay. There are no signs that are pathognomic of a diagnosis of tubercular osteomyelitis of the mandible.

Gupta et al. suggested that the presentation of such cases may often be confused with that of a pyogenic abscess or actinomycosis if sinuses are present [4]. Fukuda and Shingo, in their works on mandibular TB, suggested that the occurrence of a primary tuberculous infection in the mandible is extremely rare and in almost 43% of the cases, it is accompanied by a lesion in other regions [5]. This could also be one of the causes for the diagnostic dilemma in the present case, as there was no associated lesion elsewhere. The presentation of the waxing and waning nature of the cheek swelling, without any acute symptoms and constitutional symptoms, further added diagnostic difficulty in our present case.

Mostly, tuberculous osteomyelitis presents with pain, swelling, and, occasionally, discharge through sinuses. Mandibular involvement is very rare, as it contains less cancellous bone. However, mandibular involvement is more common than maxillary bone involvement with the alveolar and angle regions showing greater affinity [6]. A delay in diagnosis can cause significant facial deformity, a pathological fracture, temporomandibular joint involvement, and loosening and displacement of tooth buds [7]. In mandibular TB, FNAC is not as effective as an actual biopsy, but it is often adequate for diagnosis and the first choice of investigation in developing countries avoiding a surgical procedure. In our patient, FNAC yielded only hemorrhagic material, which warranted an intraoral biopsy of the lesion for diagnosis.

Sambyal SS et al. reported various radiological features of tuberculous osteomyelitis [8]. In the present patient, CECT head and neck revealed the presence of an ill-defined osteolytic lesion involving the mandibular body, suggestive of osteomyelitis. Tellez-Rodriguez et al. highlighted the clinical difficulties in establishing the etiology because of a varied spectrum of presentation of mandibular TB and proposed the use of molecular diagnostics by clinical suspicion [9].

Our patient was managed solely with ATT, as the destructive changes were reversible with medical treatment. Tellez-Rodriguez et al. performed an elective hemimandibulectomy and the reconstruction of the defect with a titanium plate in addition to antituberculosis treatment [9]. Surgery is the second option in cases with irreversible damage caused by the disease process.

## Conclusions

Mycobacterial infection as a cause of recurrent swelling of the cheek is an unusual presentation. Because of this uncommon clinical presentation, suspicion of tuberculous osteomyelitis of the mandible is difficult at the initial presentation. The treating surgeon should have a high index of suspicion of tuberculosis when an unusual cheek swelling of infective etiology not responding to antibiotic therapy. It should be considered in the differential diagnosis. Difficulties in diagnosing extrapulmonary primary TB often results in a delay in treatment, sometimes with devastating, irreversible outcomes for the patients.
